# Correction: Dolashki et al. Antimicrobial Activities of Different Fractions from Mucus of the Garden Snail *Cornu aspersum*. *Biomedicines* 2020, *8*, 315

**DOI:** 10.3390/biomedicines13030623

**Published:** 2025-03-04

**Authors:** Aleksandar Dolashki, Lyudmila Velkova, Elmira Daskalova, N. Zheleva, Yana Topalova, Ventseslav Atanasov, Wolfgang Voelter, Pavlina Dolashka

**Affiliations:** 1 Institute of Organic Chemistry with Centre of Phytochemistry, Bulgarian Academy of Sciences, Acad. G. Bonchev str., bl.9, 1113 Sofia, Bulgaria; adolashki@yahoo.com (A.D.); ventseslav.atanasov@gmail.com (V.A.); 2 Department of General and Applied, Faculty of Biology, Sofia University, St. Kliment Ohridski, Hydrobiology, 8 Dragan Tzankov Blvd., 1164 Sofia, Bulgaria; baba_emi@abv.bg (E.D.); zhelevan@phys.uni-sofia.bg (N.Z.); yanatop@abv.bg (Y.T.); 3 Interfacultary Institute of Biochemistry, University of Tübingen, Hoppe-Seyler-Straße 4, D-72076 Tübingen, Germany; wolfgang.voelter@uni-tuebingen.de

## Updated Affiliation

In the original publication [1], affiliation 2 was formatted as “Sofia University, St. Kliment Ohridski, Faculty of Biology, Department of General and Applied Hydrobiology, 8 Dragan Tzankov Blvd., 1164 Sofia, Bulgaria”. The affiliation has now been updated to follow MDPI publishing guidelines as “Department of General and Applied, Faculty of Biology, Sofia University, St. Kliment Ohridski, Hydrobiology, 8 Dragan Tzankov Blvd., 1164 Sofia, Bulgaria”.

## Error in Email Address

The email address for author Elmira Daskalova has been updated to a permanent address, baba_emi@abv.bg, replacing the one address previously listed in the original publication.

## Error in Figure

Clarification of the Error: In Figure 2, in the original publication [1], the images in positions 5 and 6 are duplicated. This error is an oversight that occurred during the preparation of the figure for the manuscript, which was not noticed by the authors. In reality, the original images corresponding to positions 5 and 6 are very similar. 

In the corrected version of Figure 2, shown below, the image in position 6 has been replaced with the correct one.

Transparency and Source Data: The authors confirm that the original figures and data files are reliably preserved and available for further verification if necessary.

Impact on the Findings: The authors declare that the scientific conclusions are not affected. This correction was approved by the Academic Editor. The original publication has also been updated.

**Figure 2:**
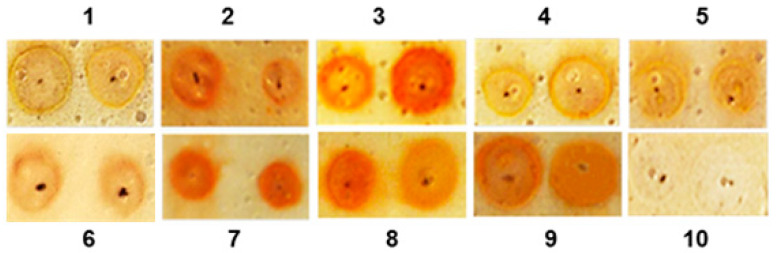
Orcinol–sulphuric acid test applied on to a silica-gel plate of different fractions isolated from the mucus of the garden snail *C. aspersum*. Spots are found in the following positions: position (1), fraction with Mw < 1 kDa; position (2), fraction with Mw < 3 kDa; position (3), fraction with Mw 3–10 kDa; position (4), fraction with Mw 5–10 kDa; position (5), fraction with Mw < 10 kDa; position (6), fraction with Mw < 20 kDa; position (7), fraction with Mw above 20 kDa; position (8), fraction with compounds of Mw > 30 kDa; position (9), fraction with compounds >50 kDa; and position (10), control, containing only water.
