# Supplementary material for: Antimicrobial Activities of Different Fractions from Mucus of the Garden Snail Cornu aspersum
Source: Biomedicines. 2020 Aug 28;8(9):315. doi: 10.3390/biomedicines8090315 (PMC7554965; doi:10.3390/biomedicines8090315)
Supplement: Supplementary file 1 [file biomedicines-08-00315-s001.pdf]

Supplementary Information No1

Peptide No1 DLTLNGLSPK

hemocyanin beta [*H. aspersa (C.aspersum)*], Sequence ID: AYO86685.1, Length: 3433, Number of Matches: 2, Score 33.3 bits(71), Identities 10/10(100%), Positives 10/10(100%), Expect 2.6, Gaps 0/10(0%)

```
Query  1    DLTLNGLSPK  10
            DLTLNGLSPK
Sbjct  303  DLTLNGLSPK  312
```

hemocyanin beta-subunit [*Helix lucorum*] Sequence ID: AEO51766.1, Length: 3433, Number of Matches: 1, Score 33.3 bits(71), Identities 10/10(100%), Positives 10/10(100%), Expect 2.6, Gaps 0/10(0%)

```
Query  1    DLTLNGLSPK  10
            DLTLNGLSPK
Sbjct  303  DLTLNGLSPK  312
```

hemocyanin beta [*Helix pomatia*] Sequence ID: AYO86688.1, Length: 3431, Number of Matches: 1, Score 33.3 bits(71), Identities 10/10(100%), Positives 10/10(100%), Expect 2.6, Gaps 0/10(0%)

```
Query  1    DLTLNGLSPK  10
            DLTLNGLSPK
Sbjct  303  DLTLNGLSPK  312
```

RecName: Full=Hemocyanin 2; AltName: Full=Keyhole limpet hemocyanin B; Short=KLH-B; Flags: Precursor [*Megathura crenulata*] Sequence ID: Q10584.2, Length: 3421, Number of Matches: 2, Score 24.8 bits(51), Identities 7/9(78%), Positives 8/9(88%), Expect 4.7, Gaps 0/9(0%)

```
Query  1    DLTLNGLSP  9
            DL LNG+SP
Sbjct  300  DLSLNGMSP  308
```

Peptide No2 MPDGALLGGGGD

APD_453|AP01405|Leptoglycin, from Leptodactylus pentadactylus [Smokey jungle frog], Antibacterial activity against Gram− bacteria: *P. aeruginosa, E. coli*, and *C. freundii*, Length=22, Score = 19.8 bits (37), Expect = 1.2, Identities = 7/8(88%), Positives = 7/8(88%), Gaps = 0/8(0%)

```
Query  4   GALLGGGG  11
           G LLGGGG
Sbjct  8   GPLLGGGG  15
```

Peptide No3 DGPADNAQGAVG

Chain A, Polyphenol oxidase, chloroplastic [*Malus domestica*] Sequence ID: 6ELS_A, Length: 506, Number of Matches: 1, Score 22.7 bits(46), Identities 8/10(80%), Positives 8/10(80%), Expect 7.0, Gaps 0/10(0**%)**

```
Query  2   GPADNAQGAV  11
           GPAD  QGAV
Sbjct  14  GPADLPQGAV  23
```

CAMPSQ1419|Linocin_M18 bacteriocin protein, UniProt: A4JQR6, *Burkholderia vietnamiensis* G4
Length=255, Score = 18.6 bits(36), Expect = 8.4, Identities = 7/12(58%), Positives = 8/12(67%), Gaps = 0/12(0%)

```
Query  1   DGPADNAQGAVG  12
           +GPA  A  AVG
Sbjct  40  EGPAGPALSAVG  51
```

Peptide No4 SLEERDIQS

hemocyanin beta [*Helix aspersa (Cornu aspersum)*], Sequence ID: AYO86685.1, Length: 3433, Number of Matches: 1, Score 31.6 bits(67), Identities 9/9(100%), Positives 9/9(100%), Expect 8.6, Gaps 0/9(0%)

```
Query  1     SLEERDIQS  9
             SLEERDIQS
Sbjct  2126  SLEERDIQS  2134
```

hemocyanin beta-subunit [*Helix lucorum*], Sequence ID: AEO51766.1, Length: 3433, Number of Matches: 1, Score 31.6 bits(67), Identities 9/9(100%), Positives 9/9(100%), Expect 8.6, Gaps 0/9(0%)

```
Query  1     SLEERDIQS  9
             SLEERDIQS
Sbjct  2126  SLEERDIQS  2134
```

hemocyanin beta [*Helix pomatia*], Sequence ID: AYO86688.1, Length: 3431, Number of Matches: 1, Score 31.6 bits(67), Identities 9/9(100%), Positives 9/9(100%), Expect 8.6, Gaps 0/9(0%)

```
Query  1     SLEERDIQS  9
             SLEERDIQS
Sbjct  2124  SLEERDIQS  2132
```

hemocyanin alphaD [*Helix aspersa (Cornu aspersum*], Sequence ID: AYO86683.1, Length: 3426, Number of Matches: 2, Score 31.6 bits(67), Identities 9/9(100%), Positives 9/9(100%), Expect 8.6, Gaps 0/9(0%)

```
Query  1     SLEERDIQS  9
             SLEERDIQS
Sbjct  2121  SLEERDIQS  2129
```

CAMPSQ5524|Maximins 31/H15, UniProt: C3RTJ3, from *Bombina microdeladigitora* [Hubei firebelly toad], Antimicrobial activity, Length=142, Score = 19.3 bits (36), Expect = 4.2, Identities = 6/7 (86%), Positives = 7/7 (100%), Gaps = 0/7(0%)

```
Query  3   EERDIQS  9
           EE+DIQS
Sbjct  21  EEKDIQS  27
```

<u>**Peptide No5   GGLLAAGAGGGGAAV**</u>

transcription factor SOX-1 [*Mesocricetus auratus*], Sequence ID: XP_005073405.2, Length: 458, Number of Matches: 1, Score42.6 bits (93), Identities 15/15(100%), Positives15/15(100%), Expect 0.003, Gaps0/15(0%)

```
Query  1    GGLLAAGAGGGGAAV  15
            GGLLAAGAGGGGAAV
Sbjct  206  GGLLAAGAGGGGAAV  220
```

APD_453|AP01405|Leptoglycin, from *Leptodactylus pentadactylus* [Smokey jungle frog], Antibacterial activity against Gram– bacteria: *P. aeruginosa, E. coli, and C. freundii*, Length=22, Score = 21.5 bits(41), Expect = 0.036, Identities = 8/12(67%), Positives = 8/12(67%), Gaps = 0/12(0%)

```
Query  1   GGLLAAGAGGGG  12
           G LL  G GGGG
Sbjct  8   GPLLGGGGGGGG  19
```

<u>**Peptide No6   LGLGNGGAGGGLVGG**</u>

CAMPSQ3913|Ctenidin-1, UniProt: P86798, from *Cupiennius salei* [Wandering spider] Antimicrobial activity, Length=119, Score = 28.0 bits(56), Expect = 0.008, Identities = 11/15(73%), Positives = 12/15(80%), Gaps = 0/15(0%)

```
Query  1   LGLGNGGAGGGLVGG  15
           +GLG GG GGGL GG
Sbjct  61  VGLGGGGYGGGLGGG  75
```

Score = 23.7 bits (46), Expect = 0.023, Identities = 11/15(73%), Positives = 11/15(73%), Gaps = 1/15(7%)

```
Query  2   GLGNG-GAGGGLVGG  15
           GLG G G GGGL GG
Sbjct  71  GLGGGQGGGGGLGGG  85
```

CAMPSQ3914 |Ctenidin-3, UniProt: P86797, from ***Cupiennius salei* [Wandering spider], Antimicrobial activity,** Length=120, Score = 28.0 bits(56), Expect = 0.008, Identities = 11/15(73%), Positives = 12/15(80%), Gaps = 0/15(0%)

```
Query  1   LGLGNGGAGGGLVGG  15
           +GLG GG GGGL GG
Sbjct  63  VGLGGGGYGGGLGGG  77
```

Score = 23.7 bits(46), Expect = 0.023, Identities = 11/15(73%), Positives = 11/15(73%), Gaps = 1/15(7%)

```
Query  2   GLGNG-GAGGGLVGG  15
           GLG G G GGGL GG
Sbjct  73  GLGGGQGGGGGLGGG  87
```

DBAASP_2872|3112|Acanthoscurrin-1, Strong activity against the Gram⁻ bacterium *E. coli* and yeast *C.albicans*, Length=132**,** Score = 26.2 bits(52), Expect = 0.033, Identities = 10/14(71%), Positives = 11/14(79%), Gaps = 0/14(0%)

```
Query  2   GLGNGGAGGGLVGG  15
           GLG GG GGG +GG
Sbjct  25  GLGGGGLGGGGLGG  38
```

Score = 26.2 bits(52), Expect = 0.033, Identities = 10/14(71%), Positives = 11/14(79%), Gaps = 0/14(0%)

```
Query  2   GLGNGGAGGGLVGG  15
           GLG GG GGG +GG
Sbjct  41  GLGGGGLGGGGLGG  54
```

DBAASP_2873|3113|Acanthoscurrin-2, Strong activity against the Gram⁻ bacterium *E.coli* and yeast *C.albicans*, Length=130**,** Score = 26.2 bits(52), Expect = 0.033, Identities = 10/14(71%), Positives = 11/14(79%), Gaps = 0/14(0%)

```
Query  2   GLGNGGAGGGLVGG  15
           GLG GG GGG +GG
Sbjct  25  GLGGGGLGGGGLGG  38
```

Score = 26.2 bits(52), Expect = 0.033, Identities = 10/14(71%), Positives = 11/14(79%), Gaps = 0/14(0%)

```
Query  2   GLGNGGAGGGLVGG  15
           GLG GG GGG +GG
Sbjct  41  GLGGGGLGGGGLGG  54
```

Peptide No7   LNLGLDAGGGDPGG

RecName: Full=Probable UDP-sugar transporter protein SLC35A4; AltName: Full=Solute carrier family 35 member A4 [Bos taurus], Sequence ID: Q05B73.1, Length: 324, Number of Matches: 1, Score 28.2 bits(59), Identities 11/13(85%), Positives 11/13(84%), Expect 0.62, Gaps1/13(7%)

```
Query  1    LNLGLDAGGGDPG  13
            LNLGL AGGG PG
Sbjct  231  LNLGLHAGGG-PG  242
```

APD_1670|AP00404|Holotricin 3, UniProt: Q25055, Antibacterial and Antifungal activity, Length = 84, Score = 21.1 bits(40), Expect = 1.3, Identities = 8/11(73%), Positives = 8/11(73%), Gaps = 0/11(0%)

```
Query  4   GLDAGGGDPGG  14
           G  AGGG PGG
Sbjct  60  GHGAGGGYPGG  70
```

AMSDb_332|CRN_LITVA|CRUSTIN, 11 kDa ATIMICROBIAL PROTEIN, CRUSTACEAN DEFENSE PROTEIN, Length=163, Score = 20.6 bits(39), Expect = 2.2, Identities = 7/11(64%), Positives = 8/11 (73%), Gaps = 0/11(0%)

```
Query  4   GLDAGGGDPGG  14
           G+  GGG PGG
Sbjct  37  GVGVGGGFPGG  47
```

Peptide No8   FNHKSLPKLEN

Chain C, Neutral trehalase [*Saccharomyces cerevisiae* S288C] Sequence ID: 5N6N_C, Length: 756, Number of Matches: 1, Score 21.8 bits(44), Identities 6/7(86%), Positives 6/7(85%), Expect 0.19, Gaps 0/7(0%).

```
Query  4    KSLPKLE  10
            K LPKLE
Sbjct  598  KALPKLE  604
```

hypothetical protein CHL9767_09410 [*Campylobacter hyointestinalis* subsp. lawsonii], Sequence ID: RAZ61964.1, Length: 59, Number of Matches: 1, Score 27.4 bits(57), Identities 9/10(90%), Positives 9/10(90%), Expect 0.21, Gaps 1/10(10%)

```
Query  3   HKSLPK-LEN  11
           HKSLPK LEN
Sbjct  39  HKSLPKWLEN  48
```

hemocyanin beta [*Helix aspersa (Cornu aspersum)*], Sequence ID: AYO86685.1, Length: 3433, Number of Matches: 1, Score 26.9 bits(56), Identities 8/11(73%), Positives 8/11(72%), Expect 0.28, Gaps 0/11(0%)

```
Query  1     FNHKSLPKLEN  11
             FNH S P LEN
Sbjct  1561  FNHQSIPQLEN  1571
```

hemocyanin beta-subunit [*Helix lucorum*] ,Sequence ID: AEO51766.1, Length: 3433, Number of Matches: 1, Score 26.9 bits(56), Identities 8/11(73%), Positives 8/11(72%), Expect 0.28, Gaps 0/11(0%)

```
Query  1     FNHKSLPKLEN  11
             FNH S P LEN
Sbjct  1561  FNHQSIPQLEN  1571
```

hemocyanin beta [*Helix pomatia*] Sequence ID: AYO86688.1, Length: 3431, Number of Matches: 1, Score 26.9 bits(56), Identities 8/11(73%), Positives 8/11(72%), Expect 0.28, Gaps 0/11(0%)

```
Query  1     FNHKSLPKLEN  11
             FNH S P LEN
Sbjct  1561  FNHQSIPQLEN  1571
```

Peptide No9   NLVGGLSGGGRGGAPGG

DBAASP_2873|3113|Acanthoscurrin-2, UniProt: Q8I6R7, Antibacterial (Gram^{-}) and Antifungal activity, Length=130, Score = 26.2 bits (52), Expect = 0.026 Identities = 10/14 (71%), Positives = 10/14(71%), Gaps = 0/14(0%)

```
Query  4    GGLSGGGRGGAPGG  17
            GGL GGG GG  GG
Sbjct  97   GGLGGGGLGGGRGG  110
```

Score = 24.5 bits (48), Expect = 0.13, Identities = 11/14(79%), Positives = 11/14(79%), Gaps = 1/14(7%)

```
Query  4    GGLSGGGRGGAPGG  17
            GGL GGGRGG  GG
Sbjct  102  GGL-GGGRGGYGGG  114
```

Score = 24.1 bits (47), Expect = 0.19, Identities = 9/13(69%), Positives = 9/13(69%), Gaps = 0/13(0%)

```
Query  4   GGLSGGGRGGAPG  16
           GGL GGG GG  G
Sbjct  29  GGLGGGGLGGGKG  41
```

DBAASP_2872|3112|Acanthoscurrin-1, Antibacterial, Antifungal, Antiparasitic activity, Length=132, Score = 26.2 bits (52), Expect = 0.026, Identities = 10/14(71%), Positives = 10/14(71%), Gaps = 0/14(0%)

```
Query  4    GGLSGGGRGGAPGG  17
            GGL GGG GG  GG
Sbjct  97   GGLGGGGLGGGRGG  110
```

Score = 25.0 bits (49), Expect = 0.097, Identities = 11/14(79%), Positives = 11/14(79%), Gaps = 1/14(7%)

```
Query  4    GGLSGGGRGGAPGG  17
            GGL GGGRGG  GG
Sbjct  102  GGL-GGGRGGGYGG  114
```

Score = 24.1 bits (47), Expect = 0.19, Identities = 9/13(69%), Positives = 9/13(69%), Gaps = 0/13(0%)

```
Query  4   GGLSGGGRGGAPG  16
           GGL GGG GG  G
Sbjct  29  GGLGGGGLGGGKG  41
```

APD_453|AP01405| Leptoglycin, from *Leptodactylus pentadactylus* [Smokey jungle frog], Antibacterial activity against Gram− bacteria: *P. aeruginosa, E. coli,* and *C. freundii*, Length=22, Score = 21.5 bits(41), Expect = 0.41, Identities = 11/19(58%), Positives = 12/19(63%), Gaps = 3/19(16%)

```
Query  2   LVGGLSG---GGRGGAPGG  17
           L+GGL G   GG GG  GG
Sbjct  2   LLGGLLGPLLGGGGGGGGG  20
```

Peptide No10   LGGLGGGGAGGGGLVGEPG

DBAASP_2873|3113|Acanthoscurrin-2, UniProt: Q8I6R7, Antibacterial (Gram−) and Antifungal activity, Length=130, Score = 34.0 bits (70), Expect = 1e-004, Identities = 14/18(78%), Positives = 14/18(78%), Gaps = 0/18(0%)

```
Query  2   GGLGGGGAGGGGLVGEPG  19
           GGLGGGG GGGGL G  G
Sbjct  24  GGLGGGGLGGGGLGGGKG  41
```

Score = 34.0 bits (70), Expect = 1e-004, Identities = 14/18(78%), Positives = 14/18(78%), Gaps = 0/18(0%)

```
Query  2   GGLGGGGAGGGGLVGEPG  19
           GGLGGGG GGGGL G  G
Sbjct  50  GGLGGGGLGGGGLGGGKG  67
```

Score = 33.1 bits (68), Expect = 2e-004, Identities = 13/15(87%), Positives = 13/15(87%), Gaps = 0/15(0%)

```
Query  2   GGLGGGGAGGGGLVG  16
           GGLGGGG GGGGL G
Sbjct  45  GGLGGGGLGGGGLGG  59
```

DBAASP_2872|3112|Acanthoscurrin-1, Antibacterial, Antifungal, and Antiparasitic activity, Length=132, Score = 34.0 bits (70), Expect = 1e-004, Identities = 14/18(78%), Positives = 14/18(78%), Gaps = 0/18(0%)

```
Query  2   GGLGGGGAGGGGLVGEPG  19
           GGLGGGG GGGGL G  G
Sbjct  24  GGLGGGGLGGGGLGGGKG  41
```

Score = 34.0 bits (70), Expect = 1e-004, Identities = 14/18(78%), Positives = 14/18(78%), Gaps = 0/18(0%)

```
Query  2   GGLGGGGAGGGGLVGEPG  19
           GGLGGGG GGGGL G  G
Sbjct  50  GGLGGGGLGGGGLGGGKG  67
```

Score = 33.1 bits (68), Expect = 2e-004, Identities = 13/15(87%), Positives = 13/15 87%), Gaps = 0/15(0%)

```
Query  2   GGLGGGGAGGGGLVG  16
           GGLGGGG GGGGL G
Sbjct  45  GGLGGGGLGGGGLGG  59
```

APD_453|AP01405|Leptoglycin, from *Leptodactylus pentadactylus*, Antibacterial activity against Gram⁻ bacteria: *P. aeruginosa, E. coli,* and *C. freundii*, Length=22, Score = 28.8 bits(58), Expect = 0.001, Identities = 13/20(65%), Positives = 14/20(70%), Gaps = 5/20(25%)

```
Query  1   LGGL-----GGGGAGGGGLV  15
           LGGL     GGGG GGGGL+
Sbjct  3   LGGLLGPLLGGGGGGGGGLL  22
```

APD_1670|AP00404|Holotricin 3, UniProt: Q25055, from *Holotrichia diomphalia* [Korean black chafer] Antibacterial and Antifungal activity, Length=84, Score=29.3 bits(59), Expect=0.003, Identities=12/18(67%), Positives=13/18(72%),Gaps=3/18(17%)

```
Query  2   GGLGGGGAGGGGLVGEPG  19
           GG GGGG+GGG   G PG
Sbjct  46  GGRGGGGSGGG---GSPG  60
```

Peptide No 11   NLVGGSGGGGRGGANPLG

APD_453|AP01405| Leptoglycin, from *Leptodactylus pentadactylus*, Antibacterial activity against Gram⁻ bacteria: *P. aeruginosa*, *E. coli*, *C. freundii*, Length = 22, Score = 22.4 bits (43), Expect = 0.21, Identities = 8/11(73%), Positives = 9/11(82%), Gaps = 0/11(0%)

```
Query  2   LVGGSGGGGRG  12
           L+GG GGGG G
Sbjct  10  LLGGGGGGGGG  20
```

APD_1398|AP02256|Procambarin, Length=155, Score = 28.4 bits(57), Expect = 0.008, Identities = 10/15(67%), Positives = 11/15(73%), Gaps = 0/15(0%)

```
Query  4   GGSGGGGRGGANPLG  18
           GG GGGG GG  P+G
Sbjct  26  GGFGGGGLGGGKPIG  40
```

CAMPSQ322|Holotricin-3, UniProt: Q25055, from *Holotrichia diomphalia* [Korean black chafer], Antibacterial and Antifungal activity, Length=84, Score = 26.7 bits(53), Expect = 0.022, Identities = 9/10(90%), Positives = 9/10(90%), Gaps = 0/10(0%)

```
Query  4   GGSGGGGRGG  13
           GG GGGGRGG
Sbjct  41  GGHGGGGRGG  50
```

Score=24.1 bits(47), Expect=0.16, Identities=9/15(60%), Positives=9/15(60%), Gaps=0/15(0%)

```
Query  4   GGSGGGGRGGANPLG  18
           GG GGGG GG    G
Sbjct  46  GGRGGGGSGGGGSPG  60
```

Peptide No 12   NGPNGGLGGSLVNGDPK

glycine-rich cell wall structural protein 1.0 isoform X4 [*Ceratitis capitata*],
Sequence ID: XP_020714877.1, Length: 388, Number of Matches: 1, Score 31.6 bits(67), Identities 13/18(72%), Positives13/18(72%), Expect 0.018, Gaps1/18(5%)

```
Query  1    NGPNGGLGGSLVNG-DPK  17
            NGPNGG  G L NG DPK
Sbjct  294  NGPNGGNSGNLPNGHDPK  311
```

Peptide No 13   GLLGGGGGAGGGGLVGGLLNG

DBAASP_2873|3113|Acanthoscurrin-2, UniProt: Q8I6R7, from *Acanthoscurria gomesiana* [Tarantula spider], Antibacterial (Gram⁻ bacteria) and Antifungal activity.
Length=130, Score = 32.7 bits(67), Expect = 3e-004, Identities = 15/21(71%), Positives = 15/21(71%), Gaps = 0/21(0%)

```
Query  1   GLLGGGGGAGGGGLVGGLLNG  21
           G LGGG G GGGGL GG L G
Sbjct  34  GGLGGGKGLGGGGLGGGGLGG  54
```

Score = 32.7 bits(67), Expect = 3e-004, Identities = 15/21(71%), Positives = 15/21(71%), Gaps = 0/21(0%)

```
Query  1    GLLGGGGGAGGGGLVGGLLNG  21
            G LGGG G GGGGL GG L G
Sbjct  86   GGLGGGKGLGGGGLGGGGLGG  106
```

Score = 31.0 bits(63), Expect = 0.001, Identities = 13/17(76%), Positives = 13/17(76%), Gaps = 0/17(0%)

```
Query  5   GGGGAGGGGLVGGLLNG  21
           GGGG GGGGL GG L G
Sbjct  43  GGGGLGGGGLGGGGLGG  59
```

CAMPSQ3894 Leptoglycin, UniProt:P86267, from *Leptodactylus pentadactylus* [Smokey jungle frog], Antibacterial activity against Gram⁻ bacteria: *P. aeruginosa, E. coli,* and *C. freundii,* Length=22, Score = 29.7 bits(60), Expect = 7e-004, Identities = 13/18(72%), Positives = 14/18 (78%), Gaps = 3/18(17%)

```
Query  1   GLLG---GGGGAGGGGLV  15
           GLLG   GGGG GGGGL+
Sbjct  5   GLLGPLLGGGGGGGGGLL  22
```

Score = 26.7 bits(53), Expect = 0.008, Identities = 15/25(60%), Positives = 15/25(60%), Gaps = 9/25(36%)

```
Query  1   GLLGG------GGGAGGGGLVGGLL  19
           GLLGG      GGG GGG   GGLL
Sbjct  1   GLLGGLLGPLLGGGGGGG---GGLL  22
```

Peptide No 14   MGGLLGGVNGGGKGGGGPGAP

APD_453|AP01405| Leptoglycin, UniProt: P86267, from *Leptodactylus pentadactylus* [Smokey jungle frog], Antibacterial activity against Gram⁻ bacteria: *P. aeruginosa, E. coli,* and *C. freundii,* Length = 22, Score = 30.6 bits (62), Expect = 4e-004, Identities = 12/17(71%), Positives = 14/17(82%), Gaps = 0/17(0%)

```
Query  1   MGGLLGGVNGGGKGGGG  17
           +GGLLG + GGG GGGG
Sbjct  3   LGGLLGPLLGGGGGGGG  19
```

Score = 24.1 bits(47), Expect = 0.062, Identities = 11/16(69%), Positives = 12/16(75%), Gaps = 2/16(13%)

```
Query  1   MGGLLGGVNGGGKGGG  16
           +G LLGG  GGG GGG
Sbjct  7   LGPLLGG--GGGGGGG  20
```

Score = 23.7 bits(46), Expect = 0.088, Identities = 11/17(65%), Positives = 12/17(71%), Gaps = 0/17(0%)

```
Query  3   GLLGGVNGGGKGGGGPG  19
           GLLGG+ G   GGGG G
Sbjct  1   GLLGGLLGPLLGGGGGG  17
```

CAMPSQ3913|Ctenidin-1, UniProt:P86798, from *Cupiennius salei* [Wandering spider], Antimicrobial activity, Length=119, Score = 30.6 bits(62), Expect = 0.002, Identities = 13/18 (72%), Positives = 13/18(72%), Gaps = 0/18(0%)

```
Query  2    GGLLGGVNGGGKGGGGPG  19
            GGL GG  GGG GGGG G
Sbjct  90   GGLGGGRGGGGYGGGGGG  107
```

Score = 27.5 bits(55), Expect = 0.016, Identities = 12/18(67%), Positives = 12/18(67%), Gaps = 0/18(0%)

```
Query  2   GGLLGGVNGGGKGGGGPG  19
           GG  GGV GG  GGGG G
Sbjct  28  GGYGGGVGGGRGGGGGLG  45
```

Score = 26.7 bits(53), Expect = 0.030, Identities = 11/16(69%), Positives = 12/16(75%), Gaps = 0/16 (0%)

```
Query  2   GGLLGGVNGGGKGGGG  17
           GG  GG  GGG+GGGG
Sbjct  65  GGGYGGGLGGGQGGGG  80
```

Peptide No 15   MLLNAKWAPHSTGPPNA

hypothetical protein AGLY_005560 [*Aphis glycines*], Sequence ID: KAE9538461.1, Length: 188, Number of Matches: 1, Score 31.2 bits(66), Identities 9/12(75%), Positives 9/12(75%), Expect 0.025, Gaps0/12(0%)

```
Query  6   KWAPHSTGPPNA  17
           KWAPH T PP A
Sbjct  79  KWAPHTTTPPHA  90
```

CAMPSQ7235 |Hymenoptaecin-1 isoform 2 precursor [Nasonia vitripennis], from *Nasonia vitripennis* [Jewel wasp], Antimicrobial activity, Length= 161, Score = 21.9 bits (42), Expect = 0.97, Identities = 6/8(75%), Positives = 6/8(75%), Gaps = 0/8(0%)

```
Query  7   WAPHSTGP  14
           W P STGP
Sbjct  40  WPPRSTGP  47
```

CAMPSQ2692 |Hymenoptaecin-1 isoform 1 precursor *Nasonia vitripennis* [Parasitic wasp], Antimicrobial activity, Length=208, Score = 21.9 bits(42), Expect = 1.0, Identities = 6/8 (75%), Positives = 6/8(75%), Gaps = 0/8(0%)

```
Query  7   WAPHSTGP  14
           W P STGP
Sbjct  39  WPPRSTGP  46
```

Score = 21.9 bits(42), Expect = 1.0, Identities = 6/8(75%), Positives = 6/8(75%), Gaps = 0/8 (0%)

```
Query  7   WAPHSTGP  14
           W P STGP
Sbjct  87  WPPRSTGP  94
```

Peptide No 16   LPFLGLVGGLLGGSVGGGGGGGGPAL

CAMPSQ3894|Leptoglycin, UniProt: P86267, from *Leptodactylus pentadactylus* [Smokey jungle frog], Antimicrobial activity against Gram$^-$ bacteria, Length=22, Score = 38.3 bits(80), Expect = 9e-007, Identities = 15/19(79%), Positives = 17/19(89%), Gaps = 0/19(0%)

```
Query  5   GLVGGLLGGSVGGGGGGGG  23
           GL+GGLLG  +GGGGGGGG
Sbjct  1   GLLGGLLGPLLGGGGGGGG  19
```

Score = 31.4 bits(64), Expect = 2e-004, Identities = 14/18(78%), Positives = 15/18(83%), Gaps = 2/18(11%)

```
Query  5   GLVGGLLGGSVGGGGGGG  22
           GL+G LLGG  GGGGGGG
Sbjct  5   GLLGPLLGG--GGGGGGG  20
```

CAMPSQ3913 |Ctenidin-1, UniProt: P86798, from *Cupiennius salei* [Wandering spider], Antimicrobial activity, Length=119, Score = 31.9 bits(65), Expect = 7e-004, Identities = 13/20(65%), Positives = 15/20(75%), Gaps = 0/20(0%)

```
Query  4   LGLVGGLLGGSVGGGGGGGG  23
           +GL GG  GG +GGG GGGG
Sbjct  61  VGLGGGGYGGGLGGGQGGGG  80
```

Score = 29.7 bits(60), Expect = 0.004, Identities = 12/16(75%), Positives = 12/16(75%), Gaps = 0/16(0%)

```
Query  8   GGLLGGSVGGGGGGGG  23
           GG  GG VGGG GGGG
Sbjct  27  GGGYGGGVGGGRGGGG  42
```

Score = 28.8 bits(58), Expect = 0.007, Identities = 12/16(75%), Positives = 12/16(75%), Gaps = 0/16(0%)

```
Query  8    GGLLGGSVGGGGGGGG  23
            GG LGG  GGGG GGG
Sbjct  89   GGGLGGGRGGGGYGGG  104
```

Peptide No17   DVESLPVGGLGGGGGGAGGGGLVGGNLGGGAG

DBAASP_2873|3113|Acanthoscurrin-2, Strong activity against the Gram$^-$ bacterium *E.coli* and yeast *C. albicans*, Length=130, Score = 44.8 bits(95), Expect = 4e-008, Identities = 19/26(73%), Positives = 20/26(77%), Gaps = 0/26(0%)

```
Query  7   VGGLGGGGGGAGGGGLVGGNLGGGAG  32
           +GG G GGGG GGGGL GG LGGG G
Sbjct  42  LGGGGLGGGGLGGGGLGGGGLGGGKG  67
```

Score = 44.4 bits(94), Expect = 5e-008, Identities = 19/25(76%), Positives = 19/25(76%), Gaps = 0/25(0%)

```
Query  8   GGLGGGGGGAGGGGLVGGNLGGGAG  32
           GG GG GGG GGGGL GG LGGG G
Sbjct  17  GGGGGYGGGLGGGGLGGGGLGGGKG  41
```

Score = 43.9 bits(93), Expect = 7e-008, Identities = 20/27(74%), Positives = 20/27(74%), Gaps = 4/27(15%)

```
Query  8   GGLGGG----GGGAGGGGLVGGNLGGG  30
           GGLGGG    GGG GGGGL GG LGGG
Sbjct  34  GGLGGGKGLGGGGLGGGGLGGGGLGGG  60
```

CAMPSQ3913|Ctenidin-1, UniProt: P86798, from *Cupiennius salei* [Wandering spider], Antimicrobial activity, Length=119, Score = 38.9 bits(89), Expect = 8e-007, Identities = 18/26(69%), Positives = 19/26(73%), Gaps = 1/26(4%)

```
Query  8   GGLGGG-GGGAGGGGLVGGNLGGGAG  32
           GG GGG GGG GGGG +GG  GGG G
Sbjct  28  GGYGGGVGGGRGGGGGLGGGRGGGGG  53
```

Score = 38.5 bits(88), Expect = 1e-006, Identities = 18/25(72%), Positives = 18/25(72%), Gaps = 2/25(8%)

```
Query  8    GGLGGGGGGAGGGGLVGGNLGGGAG  32
            GG GGGGGG GGG  VGG  GGG G
Sbjct  21   GGYGGGGGGYGGG--VGGGRGGGGG  43
```

Score = 37.7 bits (86), Expect = 2e-006, Identities = 16/23(70%), Positives = 16/23(70%), Gaps = 0/23(0%)

```
Query  8     GGLGGGGGGAGGGGLVGGNLGGG  30
             GG GGGG G GGGG  GG  GGG
Sbjct  94    GGRGGGGYGGGGGGYGGGKYGGG  116
```

APD_453|AP01405|Leptoglycin, antibacterial activity against Gram- bacteria

Length=22, Score = 29.3 bits(59), Expect = 0.002, Identities = 13/20(65%), Positives = 15/20(75%), Gaps = 3/20(15%)

```
Query  7    VGGLGG---GGGGAGGGGLV  23
            +GGL G   GGGG GGGGL+
Sbjct  3    LGGLLGPLLGGGGGGGGGLL  22
```

Score = 22.8 bits(44), Expect = 0.39, Identities = 9/13(69%), Positives = 10/13(77%), Gaps = 0/13(0%)

```
Query  20   GGLVGGNLGGGAG  32
            GGL+G  LGGG G
Sbjct  4    GGLLGPLLGGGGG  16
```

Score = 22.4 bits (43), Expect = 0.57, Identities = 8/11(73%), Positives = 9/11(82%), Gaps = 0/11(0%)

```
Query  6    PVGGLGGGGGG  16
            P+ G GGGGGG
Sbjct  9    PLLGGGGGGGG  19
```

CAMPSQ3973|Glycine-rich protein GWK, Cucumis melo [Muskmelon], Antifungal actiwity

Length=36, Score = 38.3 bits (80), Expect = 1e-006, Identities = 17/25 (68%), Positives = 17/25(68%), Gaps = 0/25(0%)

```
Query  8    GGLGGGGGGAGGGGLVGGNLGGGAG  32
            GG GGGGG  GGGG  GG  GGG G
Sbjct  6    GGWGGGGGWKGGGGGGGGWKGGGGG  30
```

Score = 31.9 bits(65), Expect = 2e-004, Identities = 15/22(68%), Positives = 15/22(68%), Gaps = 2/22(9%)

```
Query  9    GLGGGGGGAGGGGLVGGNLGGG  30
            G GGGGGG  GGG  GG  GGG
Sbjct  16   GGGGGGGGWKGGG--GGGKGGG  35
```

APD_2350|AP02350|Microcin B, Antimicrobial activity

Length=42, Score = 36.6 bits(76), Expect = 6e-006, Identities = 17/24(71%), Positives = 18/24 (75%), Gaps = 2/24 8%)

```
Query  9    GLGGGGGGAGGGGLVGGNLGGGAG  32
            G+GGGGGG GGGG  GG  GGG G
Sbjct  2    GIGGGGGG-GGGGSCGGQ-GGGCG  23
```

Score = 31.4 bits(64), Expect = 4e-004, Identities = 19/38(50%), Positives = 21/38 55%), Gaps = 12/38(32%)

```
Query  7    VGGLGGGGGGA-----GGG------GLVGGNLG-GGAG  32
            +GG GGGGGG      GGG      G  GGN G GG+G
Sbjct  3    IGGGGGGGGGGSCGGQGGGCGGCSNGCSGGNGGSGGSG  40
```

Score = 29.6 bits(65), Expect = 0.001,Identities = 14/23(61%), Positives = 15/23(65%), Gaps = 1/23(4%)

```
Query  7    VGGLGGGGGGAGGGGLVGGNLGG  29
            +GG GGGGGG G  G  GG  GG
Sbjct  3    IGG-GGGGGGGGSCGGQGGGCGG  24
```

Supplementary Information No2

/a/ 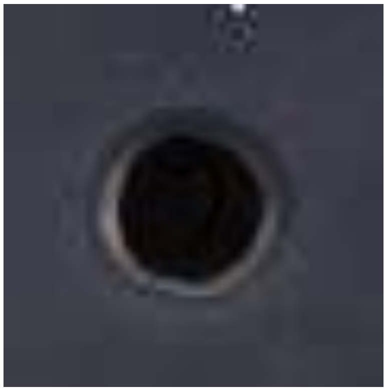 /b/ 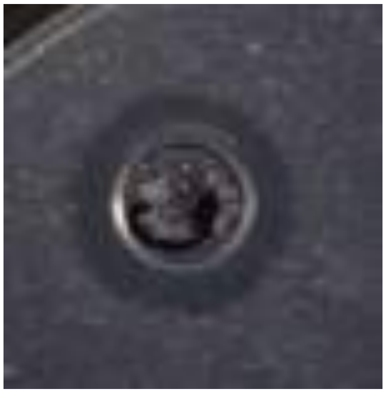

SI2, Figure s1. Illustrations of sterile areas in investigation of antibacterial activity against model bacteria antibacterial effect of Sample 4 against *Pseudomonas aureofaciens AP9* at deep aerobic cultivation /a/ - control; /b/ sterile zone for sample 4;

/a/ 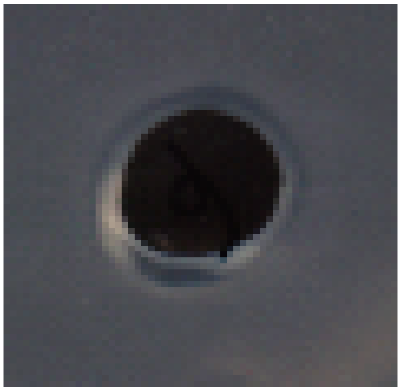 /b/ 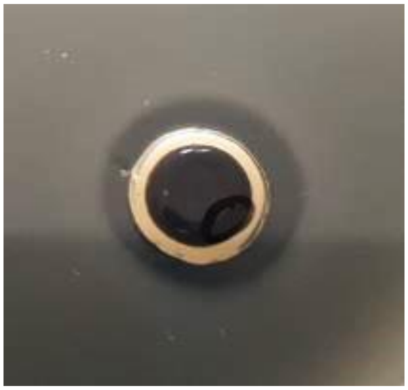

SI2, Figure s2. Illustrations of sterile areas in investigation of antibacterial activity against model bacteria antibacterial effect of Sample 4 against *Brevibacillus laterosporus BT-271* at deep aerobic cultivation /a/ - control; /b/ sterile zone for sample 4;
